# Live imaging of leukocyte recruitment in a zebrafish model of chemical liver injury

**DOI:** 10.1038/s41598-018-36771-9

**Published:** 2019-01-10

**Authors:** Michelina Stoddard, Cong Huang, Balázs Enyedi, Philipp Niethammer

**Affiliations:** 1 Tri-Institutional MD/PhD Program at Weill Cornell Medical College, New York, NY 10065 USA; 2 Cell Biology Program, Memorial Sloan Kettering Cancer Center, New York, NY 10065 USA; 3 Graduate Program in Biochemistry, Cell and Molecular Biology Allied Program, Weill Cornell Medical College, New York, NY 10065 USA; 4 Department of Physiology, Faculty of Medicine, Semmelweis University, Budapest, 1444 Hungary

## Abstract

Studying early immune responses to organ damage *in situ* requires animal models amenable to intravital imaging. Here, we used transparent zebrafish larvae, a powerful animal model for innate immunity, to measure leukocyte recruitment to damaged livers. Bath application of metronidazole (Mtz) to fish expressing nitroreductase (NTR) under a liver-specific promoter damaged the organ within 24 hours causing oxidative stress, distorted liver morphology, accumulation of TUNEL-positive cells, and transcriptional upregulation of apoptotic and antioxidant genes. Inflammatory gene transcription in damaged hepatocytes was attenuated. In line with predominant apoptosis, macrophages were massively recruited into Mtz/NTR-damaged livers. By contrast, neutrophil infiltration was more variable and delayed, consistent with less abundant necrosis and an attenuated inflammatory capacity of damaged hepatocytes.

## Introduction

Owing to their conserved innate immune system, small size, transparency, and availability in high numbers, zebrafish larvae have become a popular model for studying tissue damage-induced leukocyte recruitment by live microscopy^1–4^. Frequently used assays involve tail fin wounding with micro-knives or lasers and subsequent (non-) fluorescent imaging of leukocyte recruitment^3,4^. All these experiments imply a mechanical insult to an epithelial barrier. Yet many pathological states involve internal tissue injury without external barrier damage, for instance, anoxia, chemical/drug intoxication, or viral infection. As a detoxifying organ, the liver is often exposed to cytotoxic substances. Besides ischemia-reperfusion and viral hepatitis, drug intoxication (e.g., acetaminophen, ethanol overdose) is a main cause of liver damage in humans. Cell damage types hereby vary as a function of damage insult: Acetaminophen overdose-induced liver injury, for example, is thought to mostly involve hepatocyte necrosis^5^, with some evidence for apoptosis being debated^6^. By contrast, viral hepatitis predominantly involves apoptosis^6^. Immunological responses to necrosis versus apoptosis are thought to differ: Whereas neutrophils are attracted by tissue necrosis through damage associated molecular pattern (DAMP) signaling^7^, macrophages are attracted by “find-me” signals present on apoptotic corpses^8^. The different leukocyte populations are thought to differentially promote healing, fibrosis and regeneration after injury. Their precise mechanistic contributions to these processes remain little understood.

For studying leukocyte recruitment after liver damage, zebrafish larvae are an interesting model system that enables non-invasive live imaging of disease processes^9^. In the present study, zebrafish larvae expressing the bacterial enzyme NTR under a liver-specific promoter were treated with the non-toxic pro-drug Mtz^10^. NTR reduces Mtz into a cytotoxic compound that causes cell death. This system is frequently used for studying the physiological consequences of tissue ablation in zebrafish. However, to meaningfully utilize NTR/Mtz-induced liver injury as a model for liver pathology and regeneration, it is essential to know what types of damage, and immune responses, are provoked by NTR/Mtz.

This study provides a baseline characterization of cell death and leukocyte recruitment to NTR/Mtz-induced liver injury in live zebrafish larvae. It should facilitate further research on liver inflammation and regeneration using this popular method.

## Results

### The NTR/Mtz-system induces selective damage in the larval zebrafish liver

**Figure 1:**
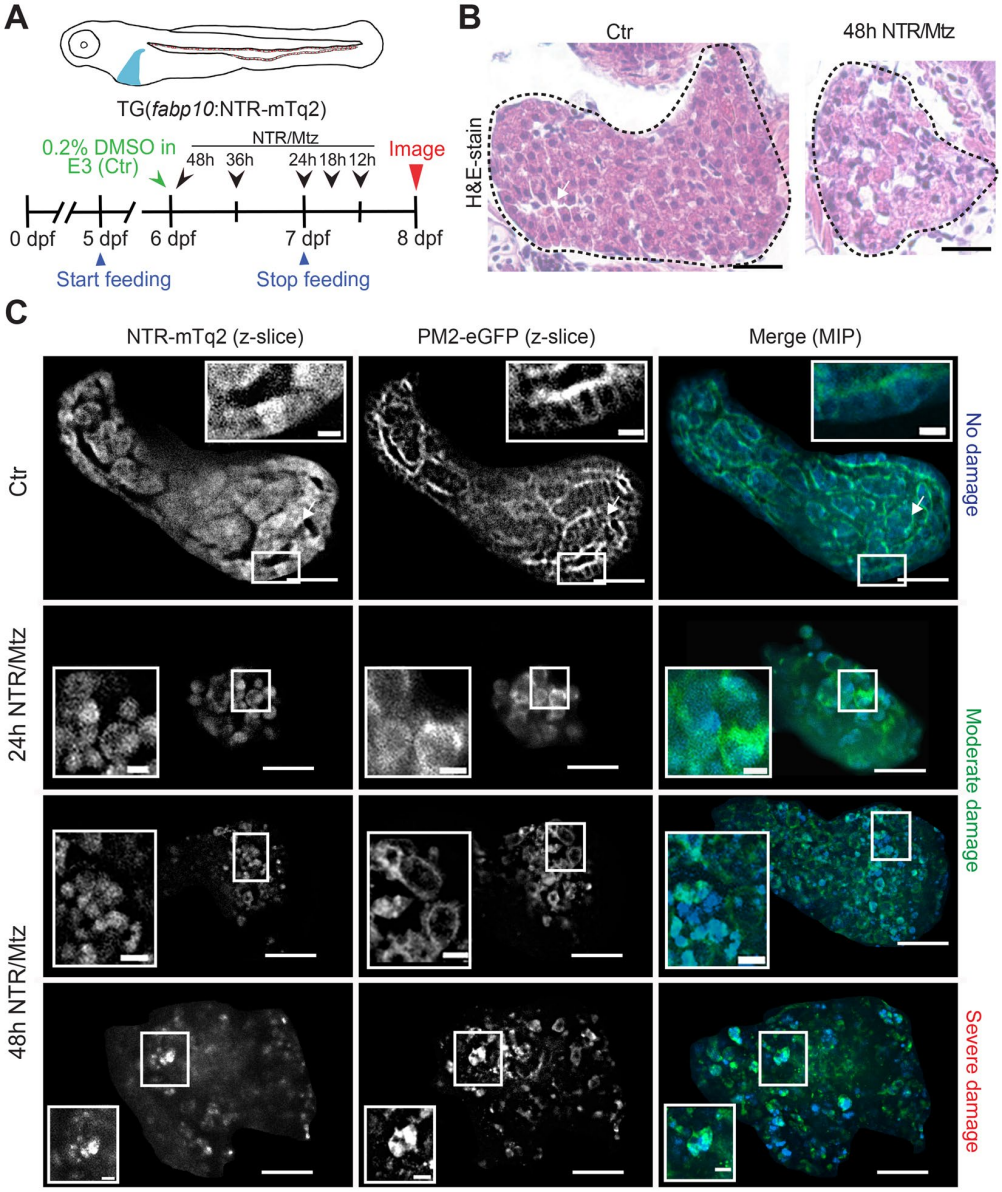
The NTR/Mtz system induces selective liver damage in larval zebrafish. (**A)** Upper panel, cartoon of transgenic larva expressing cyan fluorescent NTR fusion protein in the liver. Lower panel, scheme of experimental timeline. Black arrows/numbers indicate how long fish were bathed in 5 mM Mtz or DMSO carrier prior to live imaging. Blue arrows indicate time window of larval feeding. Feeding was stopped one day prior to imaging to reduce autofluorescence. **(B)** Haemotoxylin & Eosin (H&E) staining of 8 dpf TG(*fabp10*:NTR-mTq2; *casper*) larvae exposed to 0.2% DMSO (Ctr) or 5 mM Mtz (48 h NTR/Mtz). The “Ctr” image is representative for the H&E staining pattern in 4 of 5 larvae. The “48 h NTR/Mtz” image is representative for the H&E staining pattern in 3 of 4 larvae. Dashed line, liver outlines. Scale bars, 50 μm. **(C)** TG(*fabp10*:NTR-mTq2; *fabp10*:PM2-eGFP-P2A-mTq2-NES) larvae reveal morphological abnormalities after Mtz exposure. Representative confocal z-slices of hepatocyte cytoplasm (NTR-mTq2, cyan fluorescence, left column) or plasma membranes (PM2-eGFP, green fluorescence, middle column) highlight liver morphology. Maximum intensity projections (MIP, right column) of confocal z-slices show overall liver shape and morphology in both channels. Scale bars, 50 µm. Top row: No/Mild damage in control. Insets, regularly arranged hepatocytes with distinct cell boundaries are in close contact. Inset scale bars, 10 μm. Middle rows: Moderate damage in larvae exposed to Mtz for either 24 h (second row) or 48 h (third row). Inset, rounded hepatocytes that have lost close cell-cell contact. Inset scale bars, 10 μm. Bottom row: Severely damaged liver in larva treated with Mtz for 48 h. Insets, severely disturbed hepatocyte morphology. Scale bars, 10 μm.

**Figure 2:**
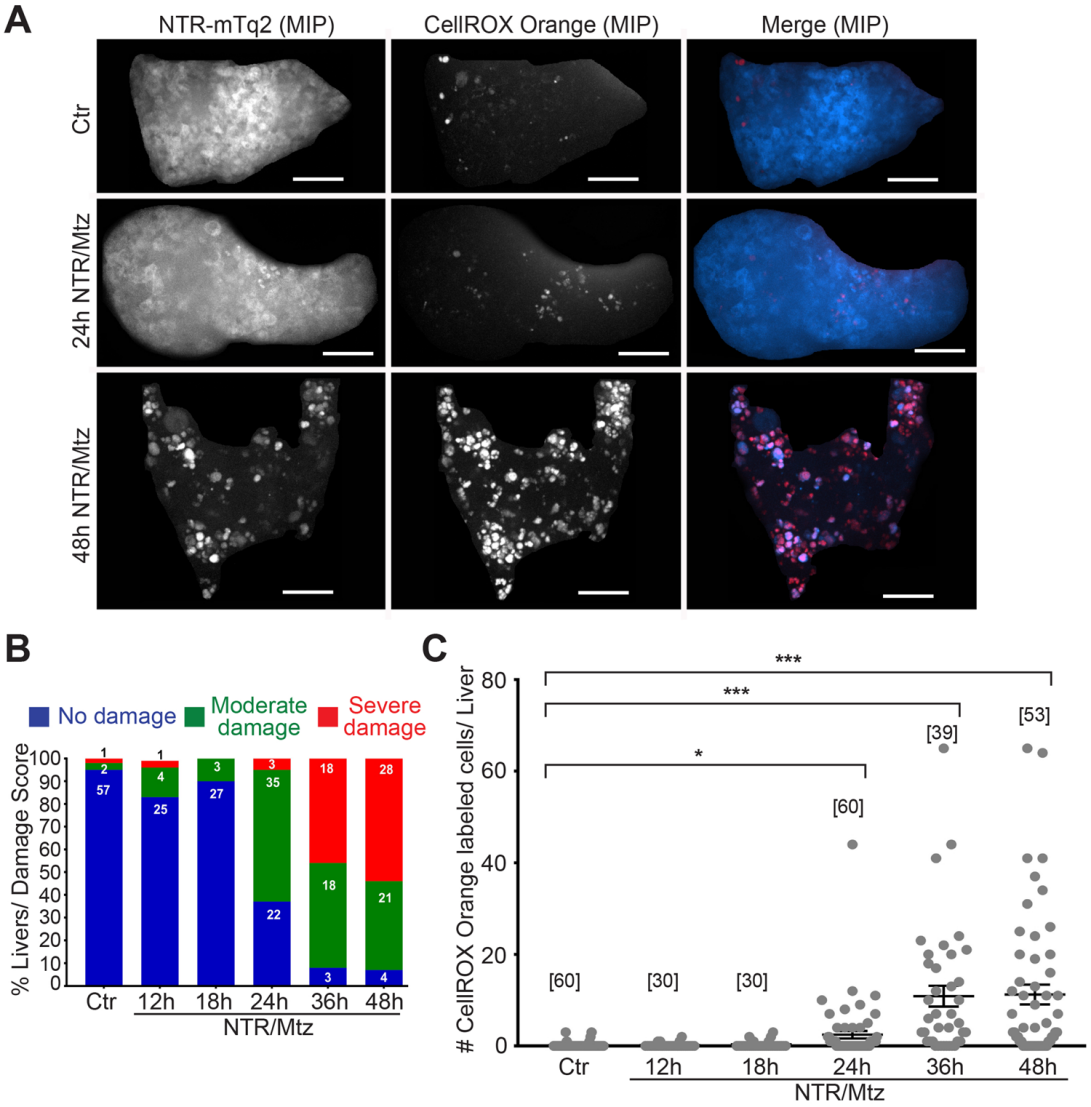
NTR/Mtz-induced liver damage is associated with oxidative stress. (**A)** Representative maximum intensity projections (MIP) of 8 dpf TG(*fabp10*:NTR-mTq2; *casper*) zebrafish livers exposed to Mtz for 24 h or 48 h. Cyan, NTR-mTq2. Red, CellROX oxidative stress dye. Scale bars, 50 µm **(B)** Quantification of morphological phenotypes by damage score. Numbers within bars indicate number of animals per group. **(C)** Quantification of CellROX live stain. Square brackets, number of animals per condition. Error bars, SEM. *t-test p < 0.05. ***t-test p < 0.0005.

We used the liver-specific *fabp10*^11^ promoter to transgenically express a C-terminal NTR fusion with mTurqoise2 (mTq2) in hepatocytes as described^10,12^ (Fig. 1A). To image fluorescent leukocytes, the *fabp10:*NTR-mTq2 transgenic line was crossed to leukocyte reporter lines with fluorescent transgenes controlled by neutrophil- (*lysC*^13^, *mpx*^3,14^), or macrophage-specific (*mpeg1*^15^) promoters. Larvae were ventrally oriented in customized agarose molds and imaged on an upright spinning disk microscope equipped with a long working-distance, water-immersion objective. With this setup, one can image up to ~100 μm deep into a transparent larva, capturing roughly 50% of the liver at this stage. NTR-mTq2 fluorescence became visible ~4 days post fertilization (dpf). Characteristic morphologic liver features, such as hepatic sinusoids and columns of rectangular, uniformly-shaped hepatocytes became visible ~5 dpf by liver histology and confocal imaging (Fig. 1B,C). 5 mM Mtz was applied by bathing at 6 dpf, that is, one day after zebrafish larvae begin to feed and livers become functional^16,17^. At 7 dpf, that is, one day after initial Mtz application, altered liver morphology manifested as progressive disruption of hepatic sinusoids, cell rounding, loss of cell-cell contacts (Fig. 1C; insets), and reduction of liver volume (Fig. S1A, left panel). CellROX Orange staining showed that progressive liver damage was accompanied by an increase in oxidative stress (Fig. 2), likely in damaged hepatocytes or recruited immune cells. The NTR/Mtz-induced morphological changes required liver expression of NTR. No altered liver morphology was observed when fish were exposed to Mtz in the absence of NTR expression (Fig. S1B). Besides reduced liver volume (Fig. S1C, red arrows) and swim-bladder deflation (Fig. S1C, blue arrows), overall morphology appeared largely normal in Mtz-treated, NTR-expressing larvae confirming tissue selectivity of this injury method^12^.

### NTR/Mtz predominantly induces hepatocyte apoptosis

To determine how hepatocytes die during NTR/Mtz-induced liver ablation, we performed terminal deoxynucleotidyl transferase dUTP Nick-End Labeling (TUNEL) to mark apoptotic cells^18^ and SYTOX Green^19^ labeling to mark necrotic cells. We noticed that damaged livers exhibited a considerable amount of red-shifted autofluorescence within small granule-like structures, perhaps representing lipofuscin, or other indigestible remnants of lysosomal degradation^20^. To exclude these structures from analysis, optimal size and intensity thresholds were empirically determined and applied as needed.

**Figure 3:**
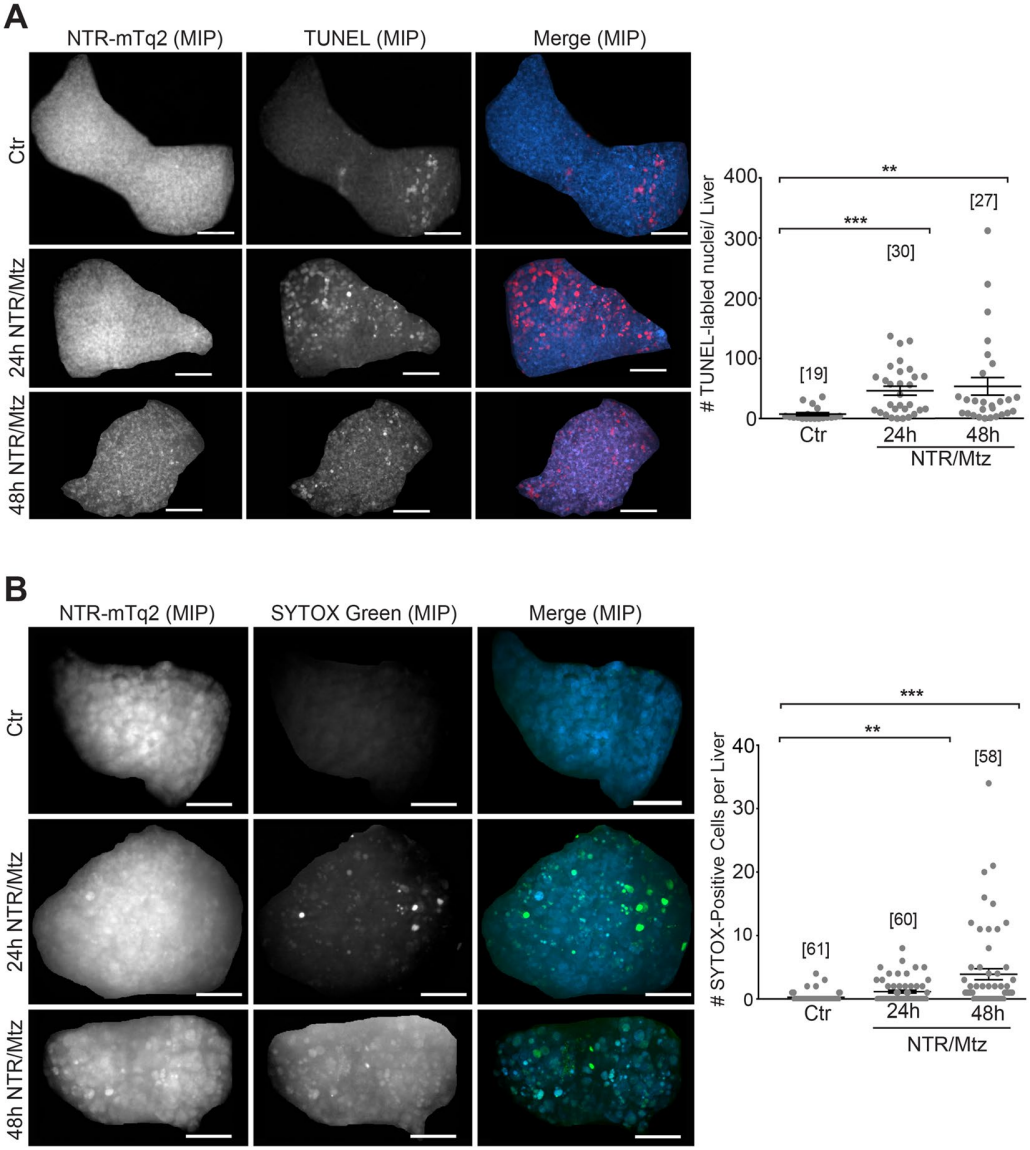
NTR/Mtz primarily induces apoptosis and sporadic necrosis. (**A)** Left panel: Representative maximum intensity projections (MIP) of zebrafish livers stained for apoptotic DNA (TUNEL) after 24 h and 48 h of Mtz exposure. Cyan, NTR-mTq2. Red, TUNEL labeling. Scale bars, 50 μm. Right panel: Quantification of TUNEL staining. Square brackets, number of animals per condition. Error bars, SEM. **t-test p < 0.005. ***t-test p < 0.0005. **(B)** Left Panel: Representative confocal maximum intensity projections (MIP) of livers stained for necrotic cells with SYTOX Green after 24 h and 48 h of Mtz exposure. Cyan, NTR-mTq2. Green, SYTOX Green. Scale bars, 50 µm. Right Panel: Quantification of SYTOX Green staining. Square brackets, number of animals per condition. Error bars, SEM. **t-test p < 0.005. ***t-test p < 0.0005.

**Figure 4:**
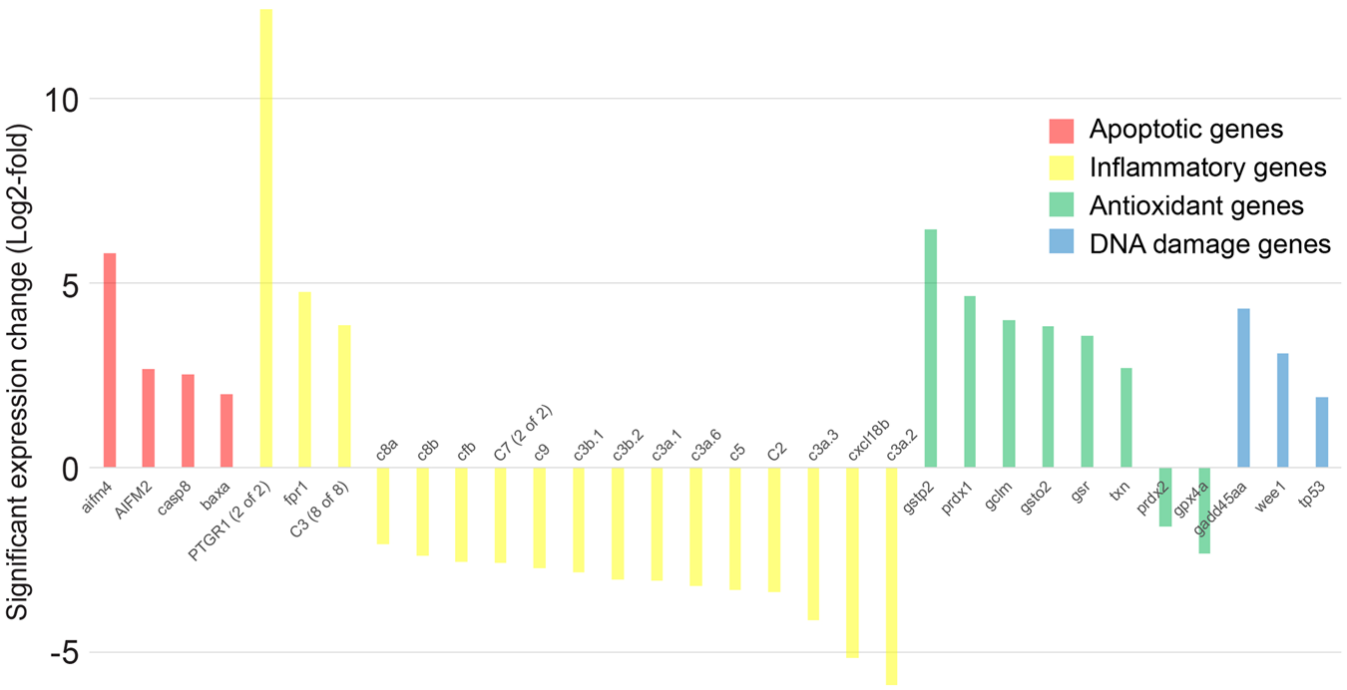
Differential expression of genes of interest in NTR/Mtz-treated hepatocytes. Hepatocytes from undamaged or damaged (48 h NTR/Mtz) zebrafish livers were FACS-sorted and analyzed for gene expression by mRNAseq. Color code highlights apoptotic (red), inflammatory (yellow), antioxidant (green), and DNA damage (blue) genes of interest. Shown are averages from two independent mRNA preparations.

After a day of Mtz exposure, TUNEL-positive cells were more abundant than SYTOX green-positive cells (Fig. 3). In line with this, mRNA sequencing of fluorescence-sorted zebrafish hepatocytes from larvae exposed to Mtz for 48 h showed upregulation of apoptosis-related genes such as *casp8*, *baxa*, *aifm2*, and *aifm4* (Fig. 4, Supplementary Table 1). We also noticed a significant upregulation of antioxidant genes, such as *prdx1*, *gsr*, *gstp1*, and *txn*, but no clear sterile inflammatory signature as would have been expected after tissue necrosis. Namely, expression of *il1b*, an important inflammatory cytokine induced by necrosis-mediated DAMP-signaling, and expression of the zebrafish chemokine *il8l1* were not significantly altered (Supplementary Table 1). Other inflammatory mediators, such as *cxcl18b*^21^, as well as complement cascade genes were downregulated (Fig. 4). Expression of *ptgr1*, a putative anti-inflammatory enzyme that inactivates the neutrophil chemoattractant LTB_4_^22^, was massively enhanced. Collectively, these data are consistent with a preponderance of a non-inflammatory cell death.

### NTR/Mtz-damaged livers primarily recruit macrophages

**Figure 5:**
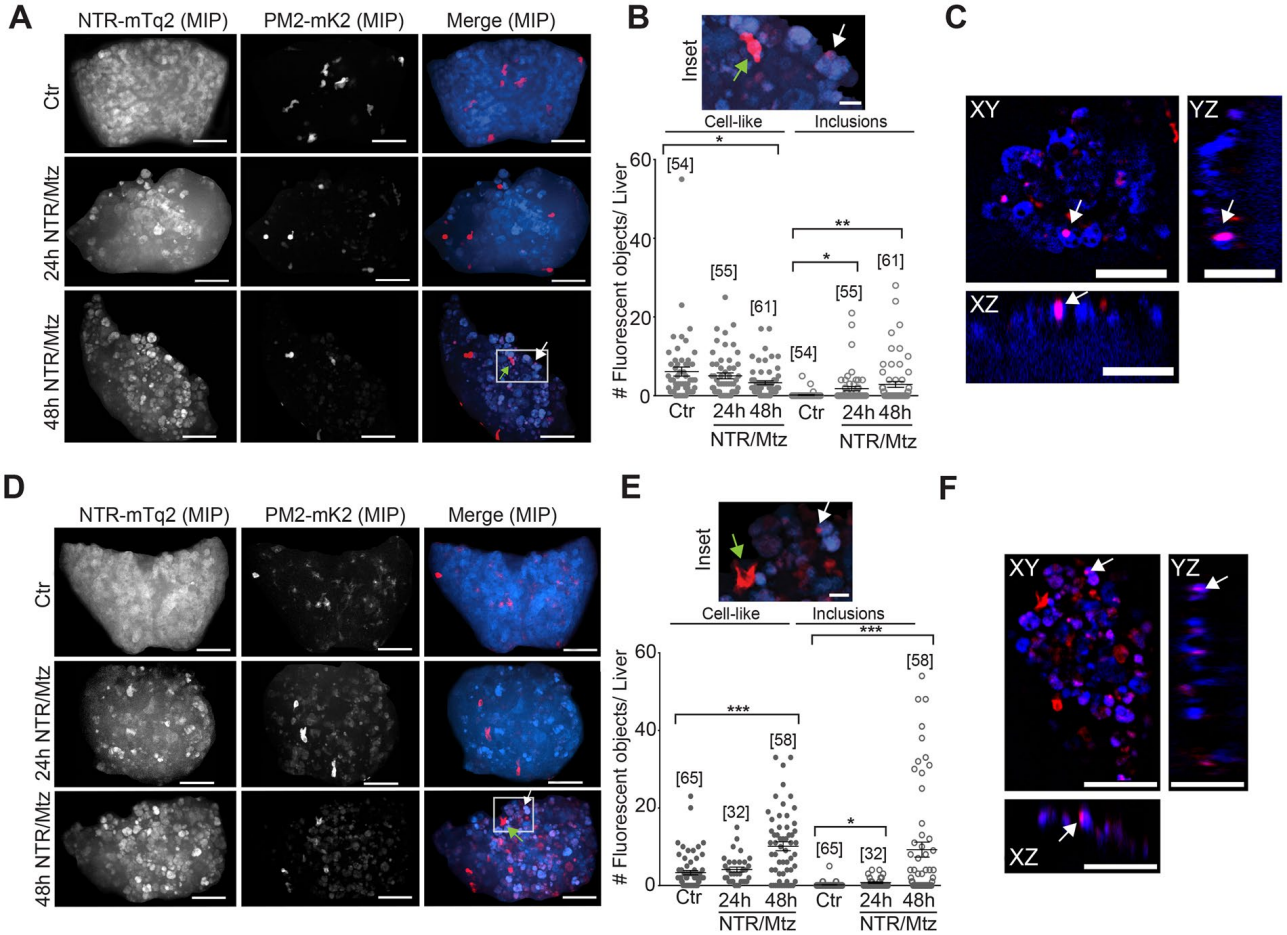
Neutrophil recruitment after Mtz-induced liver damage. (**A)** Representative confocal maximum intensity projections (MIP) of zebrafish livers with fluorescent neutrophils after indicated times of Mtz exposure in the *lysC* neutrophil reporter line. Green arrow, intact neutrophil. White arrow, red fluorescent inclusion. White box marks the inset region. Scale bars, 50 μm. **(B)** Top: Inset, magnification of marked region in A. Scale bar, 10 μm. Bottom Left: Quantification of *lysC* neutrophils in the liver at indicated times of Mtz exposure in the *lysC*:PM2-mK2 reporter line. Bottom Right: Quantification of red fluorescent inclusions in the TG(*lysC*:PM2-mK2) reporter line. Square brackets, number of animals per condition. Error bars, SEM. *t-test < 0.05. **t-test < 0.005. **(C)** Orthogonal slice view of TG(*lysC*:PM2-mK2) larval livers exposed to Mtz for 24 hours. White arrows, red fluorescent inclusions overlapping with hepatocyte. Scale bars, 50 μm. **(D)** Representative confocal maximum intensity projections (MIP) of zebrafish livers with fluorescent neutrophils after indicated times of Mtz exposure in the TG(*mpx*:PM2-mK2) reporter line. Green arrow, intact neutrophil. White arrow, red fluorescent inclusions. White box marks the inset region. Scale bars, 50 μm. **(E)** Top: Magnification of marked region in D. Green arrow, intact neutrophil. White arrow, red fluorescent inclusions. Scale bar, 10 μm. Bottom Left: number of neutrophils in the liver at indicated times of Mtz exposure in the *mpx* neutrophil reporter line. Bottom Right: number of red fluorescent inclusions in the *mpx* neutrophil reporter line. Square brackets, number of animals per condition. Error bars, SEM. *t-test p < 0.05. ***t-test p < 0.0005. **(F)** Orthogonal slice view of *lysC* neutrophil reporter larvae exposed to Mtz for 48 hours. White arrows, red fluorescent inclusion. Scale bars, 50 μm.

To investigate leukocyte recruitment to damaged livers, we crossed fluorescent neutrophil and macrophage reporters (*lysC:*PM2-mK2, *mpx:*PM2-mK2, *mpeg1:*eGFP), into the *fabp10:*NTR-mTq2 background. Tissue necrosis rapidly induces recruitment of neutrophils through DAMP-signaling, whereas apoptosis attracts macrophages through “find-me”-signals. The former process is considered inflammatory, and the latter anti-inflammatory. In line with the low numbers of necrotic cells detected by SYTOX green (Fig. 3B), neither the *lysC:*PM2-mK2 nor the *mpx:*PM2-mK2 neutrophil reporter line showed significant neutrophil recruitment to the liver at 24 h (Fig. 5A,B), or earlier 12 h and 18 h timepoints, regardless of whether neutrophil numbers were normalized to liver volume or not (Fig. S2). Liver damage was little apparent before 18 h, hence damage-induced neutrophil recruitment earlier than 12 h can be considered unlikely. Interestingly, we noticed red fluorescent inclusion bodies in both neutrophil reporter lines, most prominently visible ~48 h after Mtz application. From inspection of confocal stacks, these small structures sometimes appeared to be inside hepatocytes (Fig. 5C,F). Perhaps they represent phagocytosed, red-fluorescent (mK2) membrane debris from deceased neutrophils. However, we never directly observed neutrophils undergoing cell death or vesicle shedding (Supplementary Movies 1 and 2). Likewise, NTR/Mtz treatment did not significantly increase the number of necrotic neutrophils within the liver (Fig. S2E). Therefore, the above interpretation must be taken with caution. Interestingly, 48 h after induction of liver damage, the *mpx:*PM2-mK2 but not the *lysC:*PM2-mK2 reporter line showed some accumulation of neutrophils within severely damaged livers. The reason for the discrepancy in neutrophil behavior between the reporter lines is unclear. Based on our experience with the zebrafish tail fin injury assay, we expected both lines to report on the same neutrophil populations. Instead, the present data suggest that *lysC* and *mpx* neutrophils behave differently in response to NTR/Mtz-induced liver damage (Fig. 5B,E). It is important to mention that in the absence of NTR expression, Mtz incubation decreased neutrophil numbers in undamaged livers by ~50% (Fig. S1B, right panel). This “Mtz-only” effect could explain why we observed a counterintuitive, initial reduction of liver neutrophils shortly (12 h) after NTR/Mtz damage induction. Leukocyte numbers measured in the caudal hematopoietic niche (taken as proxy for whole animal numbers, Fig. S1D) were hardly altered, arguing for a suppressive effect of Mtz on neutrophil recruitment rather than on general neutrophil abundance/development.

**Figure 6:**
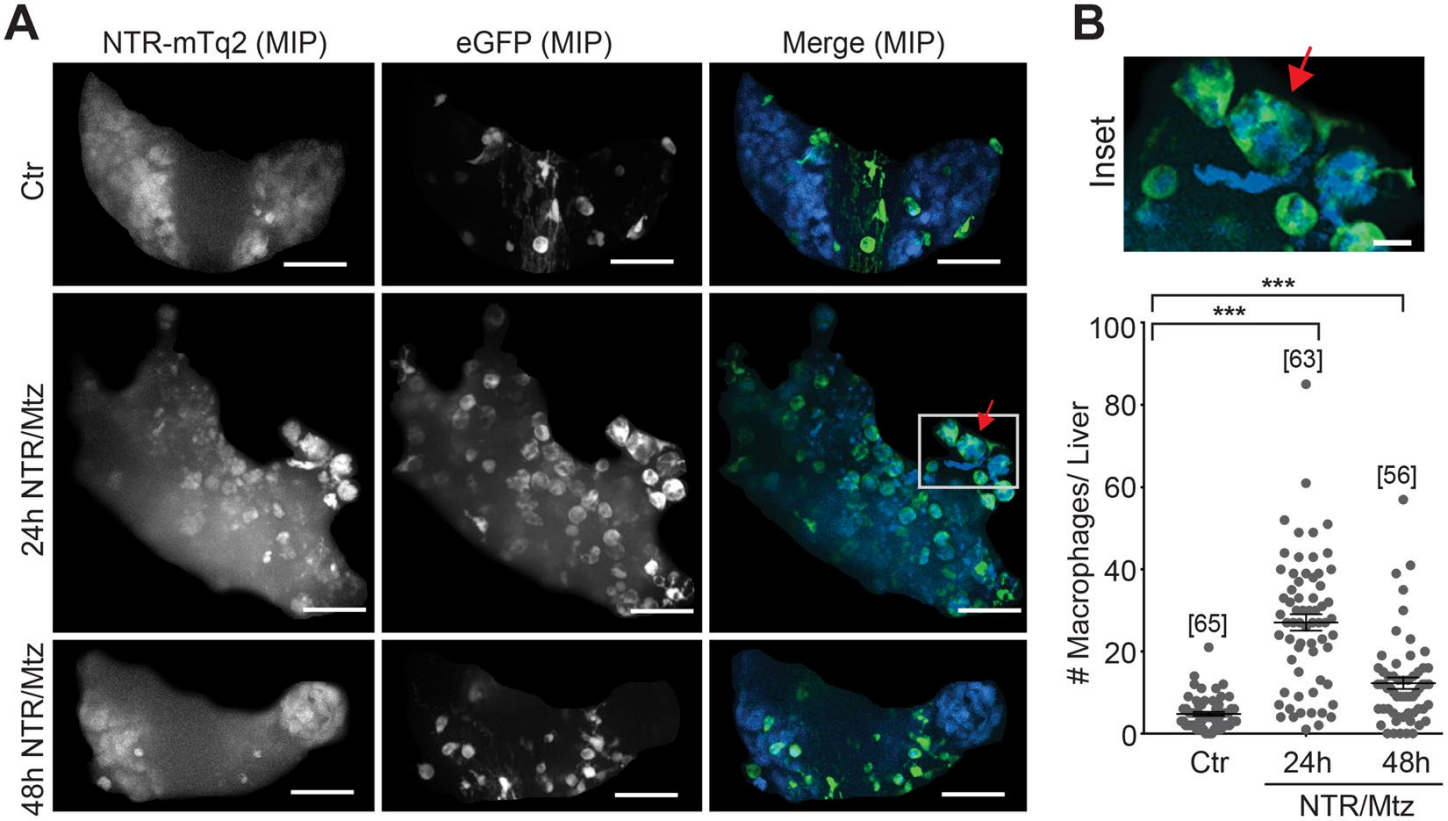
Macrophage recruitment after Mtz-induced liver damage. (**A)** Representative confocal maximum intensity projections (MIP) of zebrafish livers with fluorescent macrophages after indicated times of Mtz exposure in the *mpeg1* macrophage reporter line. Red arrow, rounded hepatocyte appears to be engulfed by a macrophage. White box, marks the inset region. Scale bars, 50 μm. **(B)** Top: Magnification of marked region in A. Scale bar, 10 μm. Bottom: Number of macrophages in the liver at indicated times of Mtz exposure in the *mpeg1* macrophage reporter line. Square brackets, number of animals per condition. Error bars, SEM. ***t-test p < 0.0005.

Unlike the ambiguous neutrophil response to liver ablation, we detected robust and early macrophage recruitment in the *mpeg1*:eGFP reporter line 24 h after damage induction (Fig. 6; Supplementary Movies 3 and 4). Prior to damage, *mpeg1*-positive macrophages mainly resided outside the liver. Only after damage did these macrophages enter the organ to engulf rounded-up, presumably apoptotic, hepatocytes (Figs 3 and 6B). Taken together, our data suggest that macrophages, and not neutrophils, are the primary responders to NTR/Mtz-induced liver injury in zebrafish larvae. Implications of these findings are discussed below.

## Discussion

NTR/Mtz-induced cell/organ ablation is widely used in zebrafish and other organisms, yet its direct immune consequences remain little studied. Here, we determine dominant cell death modes and innate immune responses to NTR/Mtz-mediated liver ablation in live zebrafish larvae. Our findings are relevant for the experimental design and interpretation of future *in vivo* studies that make use of this highly effective and popular tissue ablation method^12^.

Overall, we find that NTR/Mtz mediates liver ablation predominately through inducing hepatocyte apoptosis (Fig. 3), probably due to cytotoxic oxidative stress (Fig. 2). Upon necrotic organ damage (e.g., after physical wounding, ischemia/reperfusion- or drug-induced organ injury), the classical sequence of responses comprises initial neutrophil recruitment, followed by a second, inflammation-resolving wave of macrophages that phagocytose tissue debris and neutrophil corpses to sequester inflammatory cues. However, we did not see much evidence for early recruitment of neutrophils in our system. By measuring neutrophil recruitment at early (12 h, 18 h) timepoints, when neither necrosis nor degenerative changes to liver morphology were yet visible, we aimed to reduce the likelihood of missing such hypothetical neutrophil recruitment wave. The lack of robust neutrophil recruitment may seem odd in the light of the classic tissue damage response sequence. However, neutrophils are mainly attracted by necrosis which we only sparingly observed after NTR/Mtz-induced liver damage. Apoptosis, which dominates NTR/Mtz-induced liver ablation, is expected to attract macrophages through “find me” signals,  in line with our observations. Our data hints to some suppressive effect of Mtz on baseline neutrophil numbers in the liver. This effect may skew damage-induced leukocyte recruitment towards a pro-resolving, macrophage driven response. Such direct Mtz actions need to be carefully considered when using the NTR/Mtz system for organ regeneration studies, where type and magnitude of recruited immune cell type can critically influence outcomes (e.g., regeneration versus fibrosis).

The robust macrophage response to NTR/Mtz-induced liver damage encourages future applications of this model to dissect macrophage biology *in vivo*. Macrophages can act anti-inflammatory and pro-resolving in many ways, for example by sequestering apoptotic hepatocyte corpses before those can undergo secondary necrosis and release pro-inflammatory DAMPs. Larval zebrafish livers, like mammalian livers, are highly regenerative. Although not within the scope of the present study, we predict that NTR/Mtz-mediated liver ablation could become a valuable tool for studying macrophage functions, such as efferocytosis and M1/M2 polarization, during liver regeneration. One fascinating question is whether and how macrophages sense apoptotic liver damage from far away, because it is unclear how known “find-me signals” such as extracellular ATP or phosphatidylserine-flipping could call macrophages over longer distances^8^. Likewise, it will be intriguing to ask for the metabolic and immunological consequences of what appears to be extensive liver efferocytosis^23^. Uptake of apoptotic corpses by fly hemocytes has been proposed to generate an “innate immune memory”^24^. It will be interesting to combine the NTR/Mtz liver ablation assays with microbial infection assays^25^ to evaluate potential macrophage priming effects on innate immune defence *in vivo*.

## Methods

### Plasmid construction

A plasmid encoding the *E*.*coli* nitroreductase^26^ (NTR) enzyme (a kind gift from Dr. Mary Goll) was used as a template to PCR amplify the open reading frame of NTR, which was then fused in-frame to a DNA fragment encoding the cyan fluorescent protein mTurquoise2^27^ (mTq2). The eGFP and mKate2 (mK2) green and far-red fluorescent proteins were targeted to the plasma membrane (PM) by using the N-terminal palmitoylation/myristoylation signal of the human Lyn protein (MGCIKSKGKDSAGA, “PM2”).

To create plasmids for transgenesis using the Tol2kit system^28^, DNA fragments encoding NTR-mTq2, PM2-eGFP and PM2-mK2 were subcloned into the pME-MCS or pDONR221 middle Entry clones, respectively. The *fabp10:*NTR-mTq2 transgenesis plasmid was created by shuttling DNA sequences encoding the 2.8 kb fabp10 liver promoter^11^ (a kind gift from Dr. Laura McCormick), NTR-mTq2 and the SV40 polyadenylation sequence from Entry clones into the pDestTol2pA vector backbone with minimal tol2 elements.

The *fabp10:*PM2-eGFP-P2A-mTq2-NES and *fabp10:*PM2-eGFP-P2A-mK2 transgenesis plasmids were created for dual-color labeling of zebrafish liver cells by Gateway recombination of the *fabp10* liver promoter with the middle Entry clone containing PM2-eGFP and a p3E Entry clone containing the SV40 polyadenylation sequence on the 3’ end of the coding sequence for the viral P2A self-cleaving peptide linker^29^ fused in frame either to mK2 or mTq2-NES (subcloned from Addgene #54843^27^). The *mpx* promoter was subcloned by BamHI/KpnI digestion from a pBlueScript vector containing a 10 kb fragment of the promoter region^3^ (a kind gift from Dr. Thomas Look) into a p5E-MCS entry clone (“228”, Tol2Kit^28^). The *mpx:*PM2-mK2 transgenesis plasmid was created by LR Gateway recombination of entry plasmids containing the *mpx* promoter, PM2-mK2 and the SV40 polyadenylation sequence into the pDestTol2CG2 vector backbone with minimal tol2 elements.

### Zebrafish procedures

Zebrafish maintenance was carried out as described previously^30^ with the approval of the Institutional Animal Care and Use Committee (IACUC) of Memorial Sloan Kettering Cancer Center (MSKCC). All methods were performed in accordance with the relevant guidelines. The following wild type and previously described transgenic lines were used: *Casper*^31^ and AB (wild-type), TG*(lysC:*PM2-mK2)^32^, TG*(mpeg1:*eGFP)^15^ (Zebrafish International Resource Center). Zebrafish larvae were raised in E3 medium (5 mM NaCl, 0.17 mM KCl, 0.33 mM CaCl_2_, 0.33 mM MgSO_4_). For liver assays, larvae were raised until 5 dpf in E3, at which time they were fed AP100 Diet < 50 (Zeigler, Cat# LD50-AQ) daily until 24 hours prior to imaging.

### Reagents

The following reagents were used at indicated working concentrations: Acetone (50% in Ethanol, Sigma-Aldrich, #179124), CellROX Orange (2.5 μM in E3, Invitrogen, #C10443), Dimethyl sulfoxide (0.2% in E3, Sigma-Aldrich, #76855), Ethanol (50% in Acetone, Sigma-Aldrich, #459828), TMR Red *in situ* cell death detection kit (Sigma-Aldrich, #12156792910), low melting-point agarose (2% in E3, Gold biotechnology, #A-204-100), methanol (varying concentrations, Fisher Chemical, #A452-4), Metronidazole (5 mM in 0.2% DMSO/E3, Acros Organics, #10691063), Paraformaldehyde (4% in PBS, Sigma-Aldrich, #441244), Phosphate-buffered saline (Sigma-Aldrich, #79382), Proteinase K (10 μg/mL in PBS-T, Roche Diagnostics, #10954400), SYTOX Green (0.2 μM in E3, Invitrogen, #S7020), Tricaine (0.2 mg/mL in E3, Sigma-Aldrich, #E10521), Trypsin/EDTA (0.25% in PBS, MSKCC media core), Tween-20 (0.1% in PBS, Sigma-Aldrich, #P1379), TURBO-DNAse (4–10 U/mL in 50 mM Tris-HCL, pH 7.5, 1 mg/mL BSA, Thermo Fisher, #AM1907).

### Generation of transgenic zebrafish lines

To create transgenic lines, 25 pg of the *fabp10:*NTR-mTq2, *fabp10:*PM2-eGFP-P2A-mTq2-NES, *fabp10:*PM2-eGFP-P2A-mK2, or *mpx:*PM2-mK2 plasmid was co-injected with 25 pg of Tol2 transponase mRNA into the cytosol of one-cell stage *casper* embryos. Transgenic larvae were selected based on mosaic, liver-specific expression of the fluorescent transgene construct or heart-specific expression of eGFP driven by the cardiac myosin light chain (*cmlc*) promoter. Larvae were raised to adulthood and founders identified by crossing with wild-type fish. F1 embryos were identified by liver-specific fluorescent transgene or cardiac eGFP expression and raised to sexual maturity. Experiments were performed on progeny of F1s that were either outcrossed to *casper* adults or to the transgenic line of choice.

### Metronidazole treatment

Larvae were reared until 5 dpf in E3, at which point they were sorted for expression of the desired transgene(s) as indicated. Larvae were then transferred into a new dish containing E3 and fed larval AP100 diet (Zeigler). Larvae were incubated in 5 mM Metronidazole (Mtz, Acros Organics) in 0.2% DMSO/E3 for the indicated times (Fig. 1A). Controls were incubated in 0.2% DMSO/E3 for 48 h starting at 6 dpf. Larvae were starved for 24 h prior to imaging to suppress intestinal autofluorescence. Anesthetized (Tricaine) larvae were imaged at 8 dpf.

### Fluorescent dye labeling

TG(*fabp10:*NTR-mTq2) larvae were reared and treated with 5 mM Mtz as described above. Immediately before imaging, larvae were placed in E3 medium containing 0.2% DMSO and the specified fluorescent dye (Table 1). After dye incubation, larvae were washed 3 times with 0.2 mg/mL Tricaine in E3, embedded in low-melting point agarose, and imaged.

**Table 1:** Fluorescent Reporter Dye Concentrations and Incubation Times.

Fluorescent Dye	Final concentration (in E3)	Incubation time (min)
SYTOX Green (Invitrogen)	0.2 μM	20
CellROX Orange (Invitrogen)	2.5 μM	30

### Confocal microscopy

Experiments were performed at room temperature (~26 °C) on a Nikon Eclipse FN1 microscope equipped with a 25x Apochromat LWD NA 1.1 water immersion objective lens, a Yokogawa CSU-X1 Spinning Disk unit, an Andor iXon3 897 EMCCD camera, 405 nm, 488 nm, and 561 nm diode laser lines (Andor Revolution XD). Liver imaging experiments were performed on 8 dpf larvae anesthetized in 0.2 mg/mL Tricaine (Sigma) for 20 min before imaging. Larvae were immobilized by embedding them ventral side facing up to position the liver as close to the objective as possible. Custom agarose molds were prepared with 2% agarose dissolved in E3. Larvae were oriented within the slots of the agarose mold and layered with warm 2% low-melting point agarose (Gold Biotechnology) dissolved in E3. After the agar solidified, the imaging dish was covered with ~2–3 mL 0.2 mg/mL Tricaine in E3 to prevent desiccation and maintain anesthesia.

CellROX Orange, mK2, and tetramethylrhodamine (TMR red/TUNEL) fluorescence was excited with a 561 nm laser and sampled with a 617/73 single bandpass emission filter (Semrock). mTq2 fluorescence was excited with a 405 nm laser and sampled with a 465/30 single bandpass emission filter (Semrock). SYTOX Green and eGFP fluorescence was excited with a 488 nm laser and sampled with a 525/40 single bandpass filter (Semrock). Confocal stacks of varying thickness were captured at 2 μm z-resolution. NIS-Elements software (Nikon) was used for image acquisition.

### Image processing and analysis

All image processing tasks were completed using the programs Prism (Graphpad), Autoquant X (Media Cybernetics), Excel (Microsoft), and R. Figure 3 datasets were analyzed by two independent researchers (MS and CH). Presented figures and graphs are from the analysis conducted by MS. Results from CH were consistent within 20% of the results from MS. Files were randomly blinded prior to analysis by either researcher using a custom R script to maintain objectivity during analysis. The custom script automatically exports a master key of the original file names along with the new blinded image numbers. Blinded images were opened in Autoquant X2 as maximum intensity projections of the z-stacks and visually analyzed for morphology score (Table 2). Morphology scores for each image were recorded in an Excel spreadsheet and re-organized into a contingency plot, and Prism was used for graphical representation. Morphology scores were presented as percentages of all larvae in one treatment category (Fig. 2B).

**Table 2:** Damage Score Criteria for Liver (Confocal Imaging).

Morphology Score	Criteria
No/Mild Damage	Liver diameter up to ~200–250 μm with clearly defined organ margin. Hepatocyte diameter ~15 μm with uniform size distribution. At least 90% of cells maintain cell-cell contacts. Sinusoids are clear as spare regions flanked by intact hepatocytes. Occasional cytoplasmic clearing.
Moderate Damage	Loss of overall liver shape and distinct margins. Increased hepatocyte size. Sinusoids are hardly visible, and enlarged cells frequently show regions of cytoplasmic clearing. Loss of visible cell contacts in 10–65% of hepatocytes (rounded appearance). Appearance of “puffed” cell bodies that probably present early stages of cell death.
Severe Damage	Strongly reduced liver size and mTq2 fluorescence. Fewer than 40% of hepatocytes are intact. At least half of cells/corpses have “puffed” phenotype. Occasionally, liver margins are hard to discern.

Liver image stacks were manually cropped using the free-hand ROI tool in Autoquant X. The 3D objects counter tool was used to count fluorescent objects in these stacks. Fluorescence and size thresholds were applied to further classify these objects (e.g., as cells; Table 3). To distinguish fluorescent objects from background, a minimum intensity threshold was determined for imaging of green and red fluorescence in damaged livers. To this end, background fluorescence was measured in a region of interest in a random slice of the respective 3D stack, multiplied by 5 (or 3), and subtracted from the stack. The background multiplier was empirically selected based on prior assessment of typical liver autofluorescence in respective channels (Table 3) to ensure rigorous autofluorescence removal.

**Table 3:** Image Analysis Parameters for Confocal Liver Imaging.

Reporter/Fluorescent Dye	Minimum intensity threshold (arbitrary units)	Minimum volume (μm^3^) (desired objects counted)
*eGFP*, *mK2*	Background fluorescence X 5	70 (intact macrophages, neutrophils)
CellROX Orange	Background fluorescence X 5	10 (cell compartments with ROS)
SYTOX Green	Background fluorescence X 3	50 (hepatocyte nuclei)
TUNEL	Background fluorescence X 5	15 (apoptotic nuclei, fragmented and intact)

Volume/intensity-based object detection was manually corrected for cells lying outside the liver margin by slice-by-slice visual inspection of confocal stacks.

### TUNEL staining

The *in situ* Cell Death Detection Kit, TMR Red (Sigma-Aldrich) was used to identify apoptotic cells via the TUNEL assay. 8 dpf TG*(fabp10:*NTR-mTq2) larvae reared in either 0.2% DMSO or 5 mM Mtz for 24 h or 48 h were euthanized and fixed in 4% paraformaldehyde overnight at 4 °C. Larvae were rinsed 3 times in phosphate-buffered saline/0.1% Tween 20 (PBST) for 5 min. Larvae were dehydrated by subsequently incubating them for 5 min in each of the following solutions: (i) 25% methanol (MeOH)/75% PBST, (ii) 50% MeOH/50% PBST, (iv) 75% MeOH/25% PBST, and (v) 100% MeOH. Larvae were placed in fresh 100% MeOH and stored at −20 °C for a minimum of 12 hours. Larvae were rehydrated by subsequently incubating them for 5 min in each of the following solutions: 75% methanol (MeOH)/25% PBST, 50% MeOH/50% PBST, 25% MeOH/75% PBST. Larvae were washed 3 times with PBST, incubated in a 10 μg/mL solution of Proteinase K for 15 min, and washed 3 more times with PBST. For permeabilization, larvae were incubated in a 1:1 mixture of acetone:ethanol for 7 min at −20 °C, and were then washed 3 times with PBST. Positive controls were created by incubating control larvae in TURBO DNase (4 U/mL—10 U/mL in 50 mM Tris-HCL, pH 7.5, 1 mg/mL BSA) for 2 hours at room temperature. For each reaction, 5 μl TUNEL enzyme solution was added to 45 μl TUNEL label solution and applied to the larvae. Samples were placed overnight in a humidified chamber at 37 °C, washed 3 times with PBST, and mounted for confocal imaging.

### Extraction of hepatocyte mRNA and RNA-sequencing

Zebrafish hepatocytes for RNA-sequencing were obtained from 8 dpf larvae by fluorescence-assisted cell sorting (FACS) as follows: TG(*fabp10:*NTR-mTq2; *fabp10:*PM2-eGFP-P2A-mK2) larvae with hepatocytes expressing mTq2, eGFP, and mK2 were reared to 5 dpf as described above. Larvae were treated with 5 mM Mtz for 48 h starting at 6 dpf. Euthanized 8 dpf larvae were dissociated in 0.25% Trypsin-EDTA through mechanical disruption by pipetting. Hepatocyte sorting was carried out on a BD Aria FACS by gating for triple positive cells, expressing mTq2 and eGFP:mK2 in a 1:1 ratio. Hepatocyte mRNA was extracted using the PicoPure RNA Isolation Kit (Arcturus) and submitted to the MSKCC Genomics Core for quality analysis, SMARTer RNA amplification, and mRNA sequencing using the Illumina HiSeq System with paired-end 50 read length and an average of 20–30 million reads per sample.

The output data (FASTQ files) were mapped to the zebrafish genome GRCz10 (UCSC) using the rnaStar aligner^33^ that mapped reads genomically and resolved reads across splice junctions. A two pass -mapping method^34^ was used in which the reads were mapped twice. The first mapping pass used a list of known annotated junctions from Ensemble. Novel junctions found in the first pass were then added to the known junctions, and a second mapping pass was done (on the second pass the RemoveNoncanoncial flag was used). After mapping, the output SAM files were post processed using the PICARD tools to: add read groups, AddOrReplaceReadGroups which in additional sorted the file and converted it to the compressed BAM format. The expression count matrix was then computed from the mapped reads using HTSeq (www-huber.embl.de/users/anders/HTSeq). The raw count matrix generated by HTSeq was then processed using the R/Bioconductor package DESeq (www-huber.embl.de/users/anders/DESeq), which was used to both normalize the full dataset and analyze differential expression between sample groups.

### Statistical analysis

Statistical analysis was performed using Prism (Graphpad). Leukocyte/fluorescently labeled cell counts in each experimental condition were compared to the 0.2% DMSO in E3 controls using an unpaired, two-tailed t-test with Welch’s correction (to account for the fact that each experimental condition is not expected to have the same standard deviation).

## Electronic supplementary material

Supplementary Movie 1

Supplementary Movie 2

Supplementary Movie 3

Supplementary Movie 4

Supplementary Figures and Legends

Experimental Details for Figures

## Data Availability

The datasets generated during and/or analysed during the current study are not publicly available due to the large image file sizes but are available from the corresponding author on reasonable request.
