# Supplementary material for: Live imaging of leukocyte recruitment in a zebrafish model of chemical liver injury
Source: Sci Rep. 2019 Jan 10;9:28. doi: 10.1038/s41598-018-36771-9 (PMC6328554; doi:10.1038/s41598-018-36771-9)
Supplement: Supplementary file 5 — Supplementary Figures and Legends [file 41598_2018_36771_MOESM5_ESM.pdf]

Supplementary Information

"Live imaging of leukocyte recruitment in a zebrafish model of chemical liver injury" by Michelina Stoddard, Cong Huang, Balázs Enyedi, and Philipp Niethammer

Contains:

- Supplementary Figures
- Supplementary Figure Legends
- Supplementary Movie Legends

Figure S1

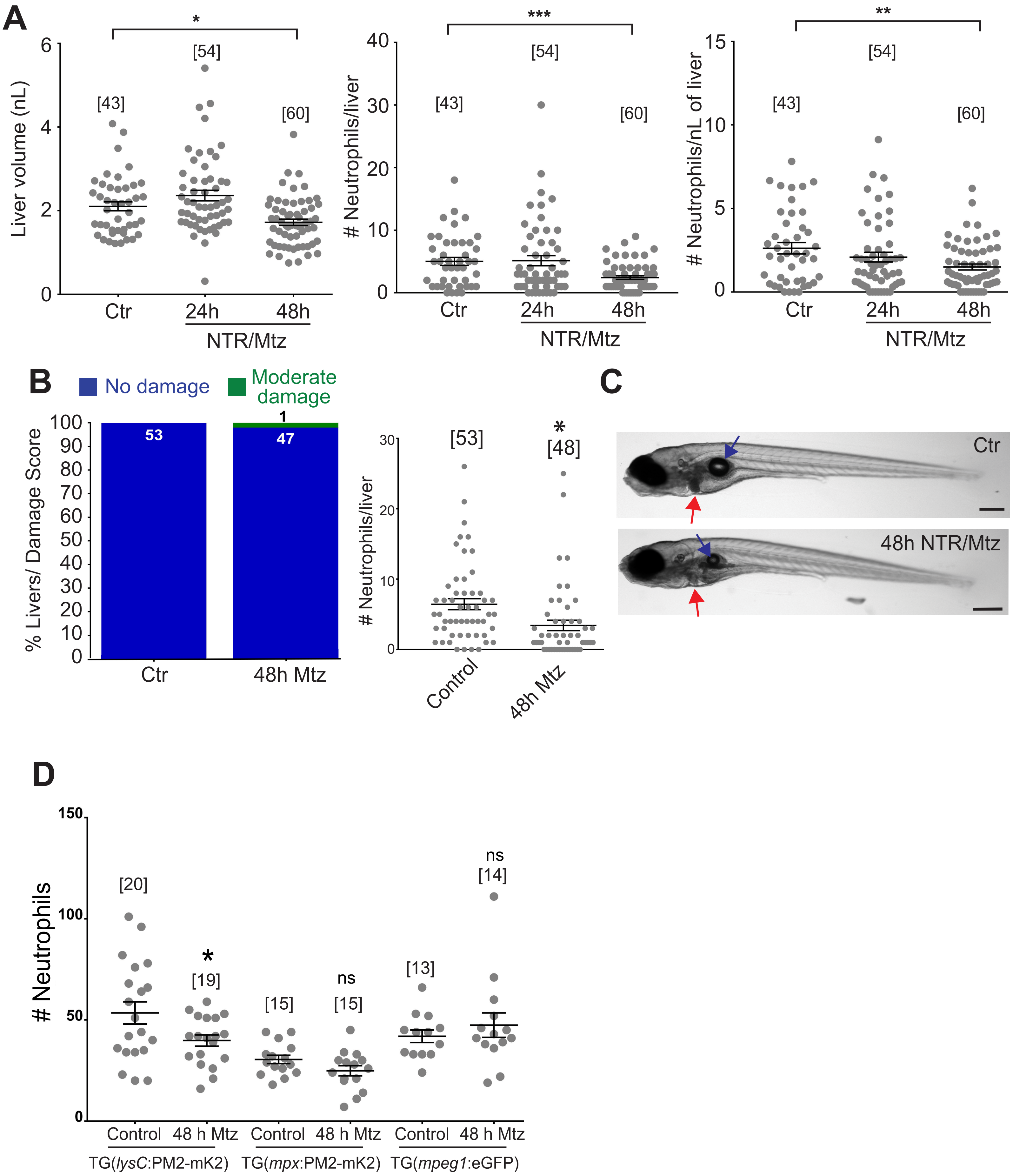
A
*
***
**
Liver volume (nL)
Neutrophils/liver
Neutrophils/nL of liver
[43]
[54]
[60]
Ctr
24h
48h
NTR/Mtz
B
No damage
Moderate damage
% Livers/ Damage Score
53
47
1
Ctr
48h Mtz
Neutrophils/liver
[53]
[48]
Control
48h Mtz
C
Ctr
48h NTR/Mtz
D
Neutrophils
[20]
[19]
[15]
[15]
[13]
[14]
ns
ns
Control
48 h Mtz
TG(lysC:PM2-mK2)
TG(mpx:PM2-mK2)
TG(mpeg1:eGFP)

Figure S2

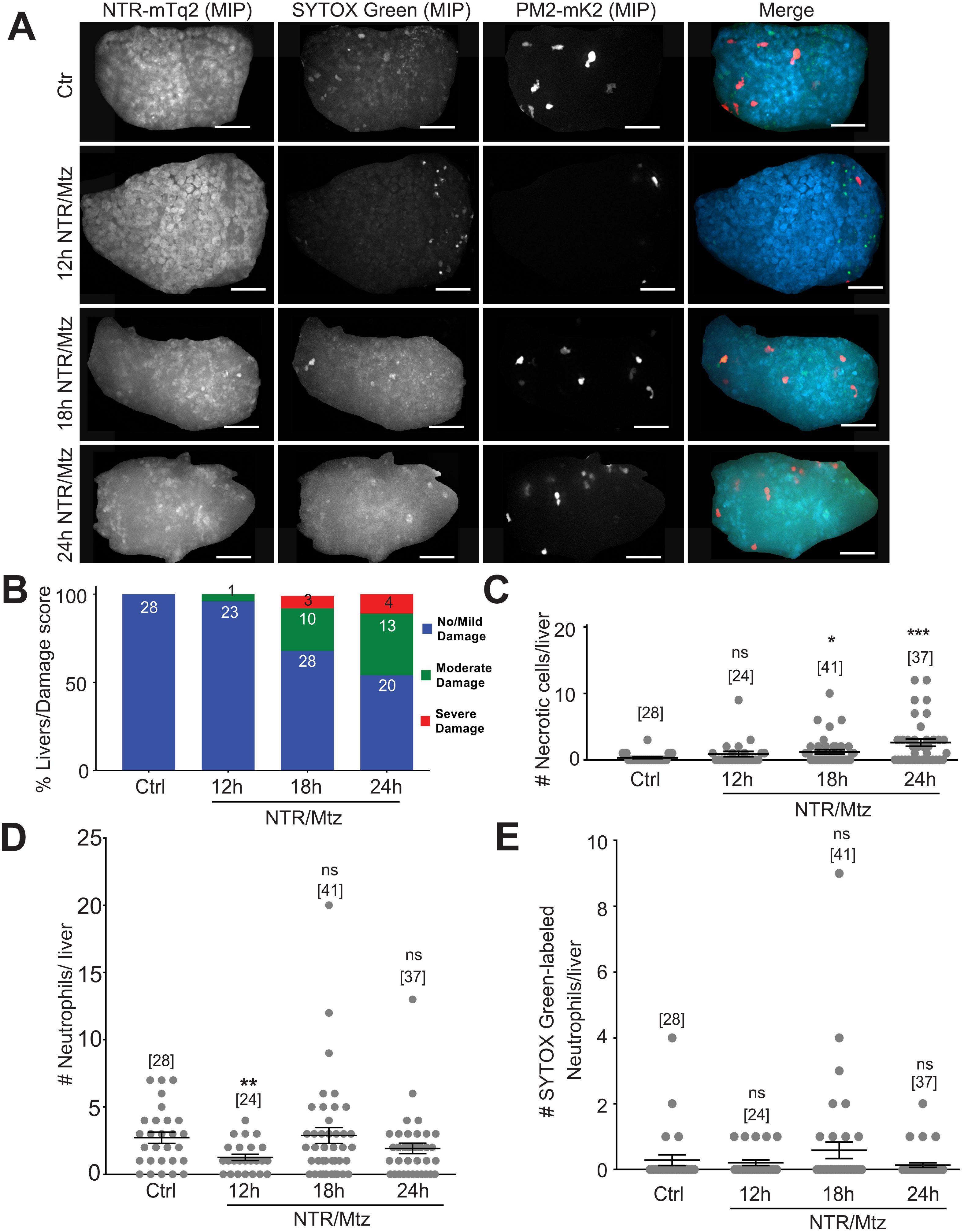
A
NTR-mTq2 (MIP)
SYTOX Green (MIP)
PM2-mK2 (MIP)
Merge
Ctr
12h NTR/Mtz
18h NTR/Mtz
24h NTR/Mtz
B
% Livers/Damage score
100
50
0
28
1
23
3
10
28
4
13
20
Ctrl
12h
18h
24h
NTR/Mtz
No/Mild Damage
Moderate Damage
Severe Damage
C
Necrotic cells/liver
20
10
0
[28]
ns
[24]
*
[41]
***
[37]
Ctrl
12h
18h
24h
NTR/Mtz
D
Neutrophils/ liver
25
20
15
10
5
0
[28]
**
[24]
ns
[41]
ns
[37]
Ctrl
12h
18h
24h
NTR/Mtz
E
SYTOX Green-labeled Neutrophils/liver
10
8
6
4
2
0
[28]
ns
[24]
ns
[41]
ns
[37]
Ctrl
12h
18h
24h
NTR/Mtz

Supplementary Figure Legends

Figure S1. (A) Left: Quantification of liver volume reduction in 8 dpf TG(*lysC*:PM-mK2; *fabp10*:NTR-mTq2) larvae treated with Mtz for 24h or 48h. Middle: Quantification of number of *lysC* neutrophils in larvae indicated above. Right: number of *lysC* neutrophils normalized by liver volume. Square brackets, number of animals per condition. Error bars, SEM. *t-test < 0.05. ***t-test < 0.0005. **(B)** Left: Quantification of liver damage by damage score in TG(*lysC*:PM2-mK2; *fabp10*:PM2-eGFP-P2A-mTq2-NES) larvae treated with Mtz for 48h. Number within bars, number of animals per damage score. Right: Quantification of *lysC* neutrophils in the livers of TG(*lysC*:PM-mK2; *fabp10*:PM-eGFP-P2A-mTq2-NES) larvae treated with Mtz for 48h. *t-test < 0.05. **(C)** TG(*lysC*:PM2-mK2; *fabp10*:NTR-mTq2) larvae show minor changes in overall morphology after 48 h Mtz exposure. Arrows, position of liver (red) and swim bladder (blue). Note reduced organ size after 48 h Mtz treatment. Scale bars, 100 μm. **(D)** Quantification of leukocytes in the tailfins of Mtz-treated TG(*lysC*:PM2-mK2; *fabp10*:NTR-mTq2) (left), TG(*mpx*:PM2-mK2; *fabp10*:NTR-mTq2) (middle), TG(*mpeg1*:eGFP; *fabp10*:NTR-mTq2) (right) larvae. Square brackets, number of animals per condition. Error bars, SEM. *t-test < 0.05.

Figure S2. (A) Representative confocal maximum intensity projections (MIP) of TG(*lysC*:PM2-mK2; *fabp10*:NTR-mTq2) larvae treated with Mtz for 12h, 18h, or 24h. Scale bars, 50 μm. **(B)** Quantification of liver damage by damage score. Numbers within bars, number of animals per condition. **(C)** Quantification of SYTOX Green-labelled (necrotic) hepatocytes in TG(*lysC*:PM2-mK2; *fabp10*:NTR-mTq2) larvae treated with Mtz for 12h, 18h, or 24h. Square brackets, number of animals per condition. Error bars, SEM.

*t-test < 0.05. ***t-test < 0.0005. **(D)** Quantification of *lysC* neutrophils in TG(*lysC*:PM2-mK2; *fabp10*:NTR-mTq2) larvae treated with Mtz for 12h, 18h, or 24h. Square brackets, number of animals per condition. Error bars, SEM. **t-test < 0.005. **(E)** Quantification of SYTOX Green-labeled (necrotic) *lysC* neutrophils in TG(*lysC*:PM2-mK2; *fabp10*:NTR-mTq2) larvae treated with Mtz for 12h, 18h, or 24h. Square brackets, number of animals per condition. Error bars, SEM.

Supplementary Movie Legends

Supplementary Movie 1. Representative confocal time-lapse movie (Z-projection) of untreated larval liver (cyan) and *lysC* neutrophils (red). Movie length, 20 min. Frame rate, 6 frames/s. Scale bar, 50 µm.

Supplementary Movie 2. Representative confocal time-lapse movie (Z-projection) of larval liver (cyan) and *lysC* neutrophils (red) after 24 hours of Mtz treatment. Movie length, 20 min. Frame rate, 6 frames/s. Scale bar, 50 µm.

Supplementary Movie 3. Representative confocal time-lapse movie (Z-projection) of untreated larval liver (cyan) and macrophages (green). Movie length, 15 min. Frame rate, 6 frames/s. Scale bar, 50 µm.

Supplementary Movie 4. Representative confocal time-lapse movie (Z-projection) of larval liver (cyan) and macrophages (green) after 24 hours of Mtz treatment. Movie length, 15 min. Frame rate, 6 frames/s. Scale bar, 50 µm.
